# Effects of Hyperbaric Oxygen Therapy on Myocardial Injury and Inflammatory Biomarkers: A Systematic Review With Qualitative Synthesis

**DOI:** 10.7759/cureus.106468

**Published:** 2026-04-05

**Authors:** Mujahed Almomany, Zaina Rawashdeh, Mohammad A Alshyyab, Abdullah Alnabulsi, Layan Alodat, Sarah A Alshamaly, Sara Aldmour, Fathi Suliman, Maya W Alsaraf, Dina M Khliefat, Joud I Alanati, Sadeen A Matar, Bader E Alsaidi, Joud Aladwan, Laith Alaswad, Lina E Alowisat

**Affiliations:** 1 General Surgery, The Jordanian Ministry of Health, Amman, JOR; 2 Faculty of Medicine, University of Jordan, Amman, JOR; 3 Family Medicine, Jordan University of Science and Technology, Irbid, JOR; 4 General Practice, Queen Alia Military Hospital, Amman, JOR; 5 Faculty of Medicine, Yarmouk University, Irbid, JOR; 6 Faculty of Medicine, Mutah University, Karak, JOR; 7 Faculty of Medicine, Hashemite University, Zarqa, JOR

**Keywords:** acute myocardial infarction, cardiac troponin, hyperbaric oxygen therapy, inflammatory biomarkers, myocardial injury, systematic review

## Abstract

Myocardial injury is a major predictor of adverse cardiovascular outcomes and is driven by both ischemic and inflammatory mechanisms. Hyperbaric oxygen therapy (HBOT), which delivers 100% oxygen at supra-atmospheric pressure, has been proposed to enhance tissue oxygenation, attenuate ischemia-reperfusion injury, and modulate inflammatory responses. However, its clinical effects on myocardial injury and inflammatory biomarkers remain unclear. This systematic review qualitatively synthesizes clinical evidence evaluating the impact of HBOT on myocardial injury and inflammatory biomarkers in adult patients. A systematic search of PubMed, Web of Science, Scopus, and the Cochrane Library through May 2025 identified randomized controlled trials and observational studies reporting myocardial injury markers (cardiac troponin I, cardiac troponin T, creatine kinase-myocardial band, creatine phosphokinase (CPK)) or inflammatory/endothelial biomarkers (C-reactive protein, high-sensitivity CRP, interleukin-6, tumor necrosis factor-alpha, endothelin-1, nitric oxide, adhesion molecules, heat shock protein 70). A meta-analysis was not performed due to substantial heterogeneity and incomplete reporting of dispersion measures. Five clinical studies including 431 patients were eligible. In acute myocardial infarction, HBOT was associated with reduced CPK levels (up to 35% reduction, p=0.03) and improved left ventricular ejection fraction (p<0.05) in randomized studies. In perioperative cardiac surgery settings, HBOT preconditioning was associated with lower postoperative inflammatory and myocardial injury biomarkers (p<0.05). In patients with chronic coronary artery disease, HBOT was linked to reductions in high-sensitivity CRP and endothelin-1 and increased nitric oxide levels (p<0.05), although one multicenter trial reported a non-significant reduction in CPK. Overall, current evidence suggests that HBOT may exert cardioprotective and anti-inflammatory effects through improved oxygen delivery and modulation of inflammatory pathways. However, available studies remain limited and heterogeneous, and larger, well-designed prospective trials are required to establish clinical benefit.

## Introduction and background

Myocardial injury represents a fundamental pathophysiological process underlying a wide range of cardiovascular conditions, including acute coronary syndromes, perioperative ischemia during cardiac surgery, and ischemia-reperfusion injury. Elevation of cardiac biomarkers, most notably cardiac troponins, is strongly associated with increased short- and long-term mortality regardless of the clinical context in which myocardial injury occurs [1]. Accordingly, myocardial injury biomarkers are widely used for both diagnosis and prognostic risk stratification.

In addition to ischemic damage, inflammatory responses play a central role in amplifying myocardial injury through cytokine-mediated apoptosis, oxidative stress, endothelial dysfunction, and maladaptive ventricular remodeling. Ischemic insult activates innate immune pathways, resulting in cytokine release and cardiomyocyte injury that contributes to microvascular impairment and progressive cardiac dysfunction [2]. Inflammatory biomarkers such as C-reactive protein (CRP), interleukin-6 (IL-6), and tumor necrosis factor-alpha (TNF-α) have been correlated with infarct size, left ventricular dysfunction, and poor prognosis following myocardial injury [3]. Consequently, therapeutic strategies that target hypoxia and inflammation are of significant clinical interest.

Cardiac biomarkers, particularly high-sensitivity troponins, are not only diagnostic tools but also powerful predictors of cardiovascular outcomes. An individual patient data meta-analysis demonstrated that high-sensitivity troponin T is independently associated with increased risk of mortality and cardiovascular events in chronic heart failure patients [4]. Similarly, inflammatory biomarkers such as high-sensitivity C-reactive protein (hs-CRP) remain strongly predictive of recurrent cardiovascular events even in statin-treated patients and are well-validated markers of residual inflammatory risk [5]. A 2025 multicenter cohort study further demonstrated the predictive value of the hs-CRP/HDL-C ratio for cardiometabolic multimorbidity [6]. Additionally, elevated IL-6 levels following STEMI have been associated with larger infarct size, reduced left ventricular ejection fraction, and adverse long-term outcomes [3]. These biomarkers reflect underlying inflammatory and ischemic processes that contribute to myocardial injury and disease progression. Therefore, therapeutic interventions that modulate these biomarkers may have clinically meaningful implications beyond biochemical changes alone.

Hyperbaric oxygen therapy (HBOT) delivers 100% oxygen under increased atmospheric pressure (typically 2.0-3.0 atmospheres absolute (ATA)), increasing dissolved plasma oxygen that results in enhanced oxygen delivery to ischemic tissue independently of hemoglobin-bound oxygen [4]. Experimental and clinical studies have demonstrated that HBOT can improve mitochondrial oxidative phosphorylation, reduce oxidative stress, suppress inflammatory cytokine production, and enhance microvascular perfusion [5]. These biological processes justify further research into the use of HBOT for treating cardiac injuries. Clinical trials assessing HBOT in cardiac conditions date back several decades, particularly in acute myocardial infarction (AMI), but reported outcomes have been inconsistent [6]. While some studies suggest reductions in infarct size and biochemical markers of myocardial damage, others report neutral clinical effects. Importantly, no comprehensive systematic review has focused specifically on myocardial injury and inflammatory biomarkers at the biomarker level using a qualitative synthesis approach. This review therefore aims to address this gap.

## Review

Methods

Study Design

This study was designed as a systematic review and conducted in accordance with the Preferred Reporting Items for Systematic Reviews and Meta-Analyses (PRISMA) 2020 guidelines.

Search Strategy and Information Sources

A comprehensive and systematic literature search was conducted in PubMed, Web of Science, Scopus, and the Cochrane Library from database inception to May 2025. The search strategy was developed a priori and combined controlled vocabulary terms (Medical Subject Headings (MeSH), where applicable) with relevant free-text keywords related to hyperbaric oxygen therapy, myocardial injury, cardiac ischemia, and inflammatory biomarkers to ensure comprehensive retrieval of eligible studies.

Eligibility Criteria

Eligible studies included randomized controlled trials and observational studies evaluating HBOT in adult patients with cardiovascular conditions and reporting myocardial injury or inflammatory biomarkers. Detailed inclusion and exclusion criteria used for study selection are summarized in Table 1 according to the PICOS (Population, Intervention, Comparison, Outcomes, and Study Design) framework.

**Table 1:** Inclusion and Exclusion Criteria for Study Selection Based on the PICOS Framework This table presents the predefined inclusion and exclusion criteria applied during study selection according to the PICOS (Population, Intervention, Comparison, Outcomes, and Study Design) framework. These criteria were used to identify relevant studies evaluating HBOT and its effects on myocardial injury and inflammatory biomarkers in adult patients with cardiovascular conditions.

Category	Inclusion Criteria	Exclusion Criteria
Study Design	Randomized controlled trials (RCTs), prospective cohort studies, retrospective observational studies	Secondary research, animal studies, in vitro research, case reports/series <10 patients, conference abstracts
Population	Adult patients (≥18 years) with cardiac conditions (AMI, stable CAD, perioperative ischemia)	Non-cardiac indications, healthy volunteers, chronic conditions not specific to the heart
Intervention	Hyperbaric oxygen therapy (HBOT) at ≥1.5 ATA with 100% oxygen	Normobaric oxygen only, or studies without HBOT evaluation
Outcomes	Myocardial injury biomarkers (cTnI, cTnT, CK-MB, CPK) OR inflammatory biomarkers (CRP/hs-CRP, IL-6, TNF-α, endothelin-1, NO, soluble adhesion molecules, HSP70)	No relevant biomarker data or absence of predefined outcomes
Other	English-language human studies with full-text availability	Abstracts-only, non-English, duplicates, insufficient methodological detail

Search Strategy and Information Sources 

A comprehensive literature search was conducted in PubMed, Web of Science, Scopus, and the Cochrane Library from database inception to May 2025. Table 2 presents the search strategy.

**Table 2:** Search Strategy by Database

Database	Search Strategy	Filters	Results
PubMed	((“hyperbaric oxygenation”[MeSH] OR “hyperbaric oxygen therapy”[tiab] OR “HBOT”[tiab]) AND (“myocardial injury”[tiab] OR “troponin”[tiab] OR “CK-MB”[tiab] OR “inflammatory biomarkers”[tiab] OR “C-reactive protein”[tiab]))	Humans; Adults; English	145
Web of Science	TS=(“hyperbaric oxygen” OR “HBOT”) AND TS=(“myocardial injury” OR “troponin” OR “inflammatory biomarker”)	English	98
Scopus	TITLE-ABS-KEY((“hyperbaric oxygen” OR “HBOT”) AND (“myocardial injury” OR “cardiac troponin” OR “inflammatory biomarker”))	English; Article	112
Cochrane Library	(“hyperbaric oxygen” OR “HBOT”) AND (“myocardial injury” OR “troponin” OR “inflammatory biomarkers”)	Trials	57

Study Selection and Data Extraction

**Figure 1:**
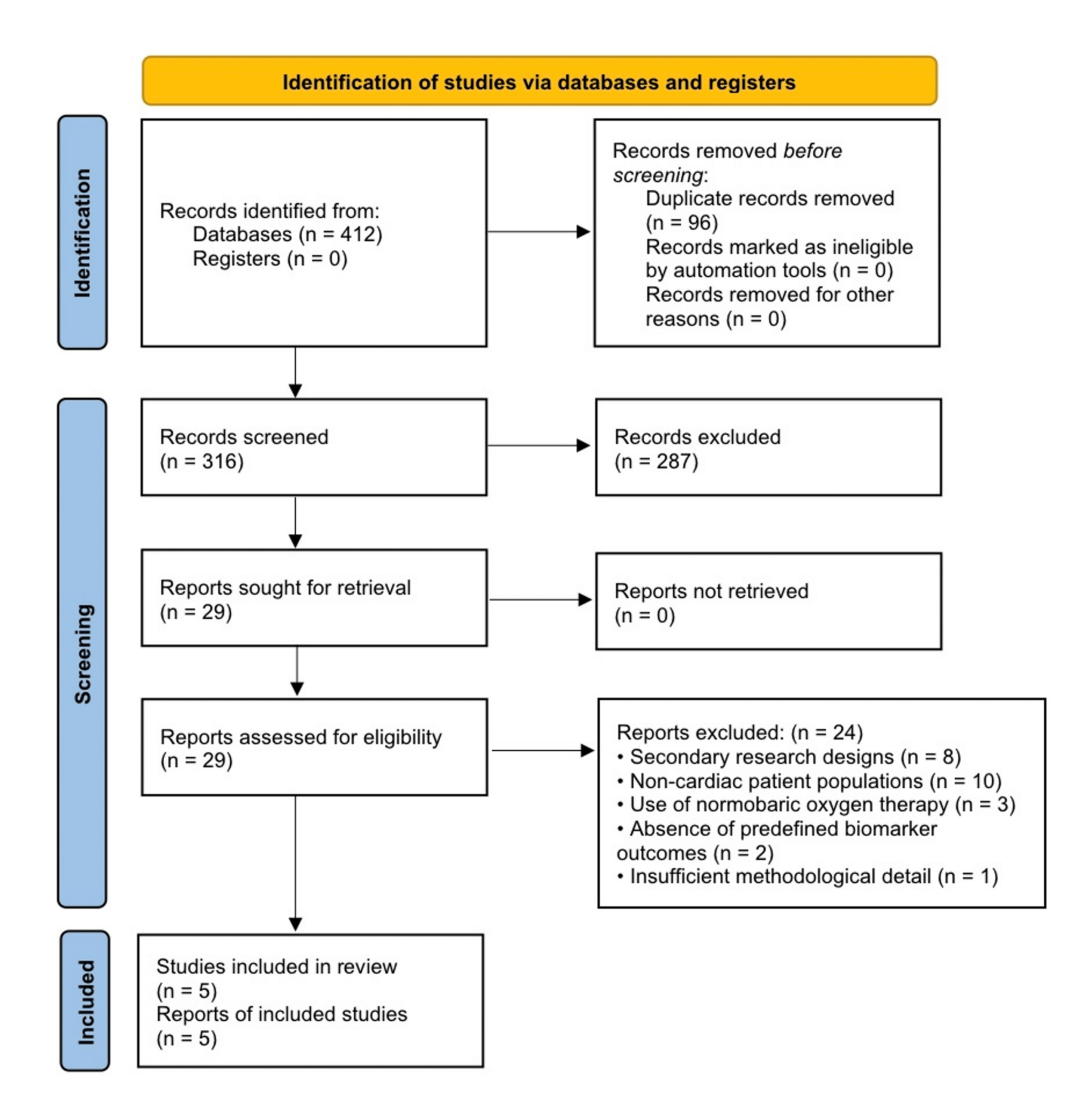
PRISMA Flow Diagram Illustrating the Study Selection Process for the Systematic Review PRISMA: Preferred Reporting Items for Systematic Reviews and Meta-Analyses

The initial database search identified 412 records. After removal of 96 duplicates, 316 studies underwent title and abstract screening, resulting in the exclusion of 287 records for irrelevance. Twenty-nine full-text articles were sought for retrieval and assessed for eligibility. Of these, 24 reports were excluded primarily due to secondary research designs, non-cardiac patient populations, use of normobaric oxygen therapy, absence of predefined biomarker outcomes, or insufficient methodological detail. A total of five studies met the inclusion criteria and were included in the qualitative synthesis. The selection process is illustrated in the PRISMA flow diagram (Figure 1).

Risk of Bias Assessment

Randomized trials were assessed using RoB 2.0. Several studies demonstrated some concerns related to allocation concealment and incomplete outcome reporting. The observational study demonstrated moderate risk of bias due to potential confounding. Table 3 summarizes the risk of bias assessment.

**Table 3:** Risk of Bias Assessment

Study	Study Design	Randomization	Deviations	Outcomes	Missing Data	Reporting	Overall
Shandling 1997 [7]	RCT pilot	Some concerns	Low	Low	Some concerns	Some concerns	Some concerns
Stavitsky 1998 [8]	RCT multicenter	Low	Low	Low	Some concerns	Some concerns	Some concerns
Dekleva 2004 [9]	RCT	Low	Low	Low	Low	Low	Low
Alex 2005 [10]	RCT	Some concerns	Low	Low	Low	Low	Some concerns
Li 2018 [11]	Observational	Moderate	Moderate	Low	N/A	N/A	Moderate

Data Analysis and Statistical Methods

We employed a structured narrative synthesis to summarize the data rather than conducting a meta-analysis. A meta-analysis was not performed because heterogeneity was judged substantial and dispersion measures were incompletely reported. Studies were first organized by clinical context (AMI vs. perioperative cardiac settings vs. chronic CAD) and then by biomarker type (myocardial injury markers such as troponins/CK-MB vs. inflammatory markers such as CRP, IL-6, and TNF-α).

We noted statistical significance when reported and indicated whether HBOT was associated with a change in biomarker levels for each paper. Effect size estimates (e.g., percentage reduction) were extracted where available. No additional statistical calculations or pooling were performed beyond the analyses conducted in the primary studies. The included studies comprised 431 patients across heterogeneous designs (range: 64-115 patients/study), limiting statistical power for definitive conclusions. Post-hoc power analysis for CPK reduction (primary outcome from largest RCT [7]; n=66, effect size d=0.8, α=0.05) yields 70% power, below conventional 80-90% thresholds typically required for clinical recommendations. This confirms the need for larger, adequately powered prospective trials.

Heterogeneity across studies was assessed qualitatively due to substantial variability in patient populations, clinical settings, HBOT protocols, and biomarker reporting. Studies were grouped by clinical context and by biomarker category to allow structured narrative comparison, without quantitative subgroup analysis. Narrative sensitivity analyses were performed by reassessing conclusions after exclusion of studies with partial eligibility, non-cardiac populations, or unclear methodology. Additional sensitivity assessment focused on studies reporting direct myocardial injury biomarkers only. A formal meta-analysis with pooled effect estimates was not feasible due to the limited number of studies, substantial clinical heterogeneity, and incomplete reporting of dispersion measures (e.g., standard deviations). Future studies with standardized outcome reporting may enable quantitative synthesis.

Ethical Considerations

Ethical approval and informed consent were not required, as this review analyzed data exclusively from previously published studies. Data extraction ensured integrity and accuracy from original sources.

Results

Study Characteristics

Five studies comprising 431 patients met inclusion criteria. These included the HOT MI pilot (n=66) [7] and multicenter (n=112) [8] trials in AMI with thrombolysis, a randomized trial in AMI (n=74) [9], a randomized trial in elective CABG with preconditioning (n=64) [10], and a retrospective observational study in CAD post-stenting (n=115) [11]. Table 4 presents study characteristics.

**Table 4:** Characteristics of the Included Studies Evaluating Hyperbaric Oxygen Therapy and Myocardial Injury or Inflammatory Biomarkers RCT: randomized controlled trial; AMI: acute myocardial infarction; CABG: coronary artery bypass grafting; CAD: coronary artery disease; HBOT: hyperbaric oxygen therapy; ATA: atmospheres absolute; CPK: creatine phosphokinase; CK-MB: creatine kinase-myocardial band; LVEF: left ventricular ejection fraction; cTnI: cardiac troponin I; hs-CRP: high-sensitivity C-reactive protein; ET-1: endothelin-1; NO: nitric oxide; CD18: cluster of differentiation 18; HSP70: heat shock protein 70

Study (Year)	Design	Population/Setting	Sample Size	HBOT Protocol (ATA; Session)	Biomarkers Measured
Shandling et al., HOT MI pilot (1997) [7]	RCT pilot	AMI + thrombolysis	66 (32 HBOT; 34 control)	HBOT adjunct to thrombolysis; ≥2 ATA; details in original	CPK/CK-MB, clinical outcomes
Stavitsky et al., HOT MI multicenter (1998) [8]	RCT multicenter	AMI + thrombolysis	112	HBOT + thrombolysis; ≥2 ATA; details in original	CPK/CK-MB, clinical outcomes
Dekleva et al. (2004) [9]	RCT	AMI after thrombolysis	74	100% O₂ at 2.0 ATA, 60 min	CPK, LV volumes, EF (numeric EF reported)
Alex et al. (2005) [10]	Double-blind RCT	Elective CABG (preconditioning)	64	100% O₂ at 2.4 ATA, two 30-min sessions preop	sE-selectin, CD18, HSP70, (troponin I mentioned)
Li et al. (2018) [11]	Retrospective observational	CAD post drug-eluting stent	115	100% O₂ at 2.0 ATA, 80 min per session (two 40-min periods); 6×/week × 4 weeks	hs-CRP, endothelin-1, NO; SPECT perfusion

Effects on Myocardial Injury Biomarkers

In the pilot HOT MI trial [7], mean CPK levels at 12-24 hours were approximately 35% lower in the HBOT group (p=0.03). Discharge LVEF was higher with HBOT (52.4% vs. 47.3%, p=NS). In the multicenter trial [8], mean CPK was 7.5% lower with HBOT (p=NS), with LVEF of 51.7% vs. 48.4% (p=NS). This non-significant finding should be interpreted cautiously and does not support a definitive conclusion of HBOT benefit on CPK reduction. Dekleva et al. [9] reported significant LVEF improvement with HBOT (46.27% to 50.81%) versus decline in controls (45.54% to 44.05%, p<0.05), with 35.3% lower peak CPK. Alex et al. [10] found significantly lower postoperative cTnI with HBOT preconditioning (p<0.05). Li et al. [11] found significant reductions in hs-CRP and endothelin-1, and increased NO with HBOT (p<0.05). Table 5 summarizes biomarker outcomes.

**Table 5:** Effects of Hyperbaric Oxygen Therapy on Myocardial Injury and Inflammatory Biomarkers: Direction and Statistical Outcomes CPK: creatine phosphokinase; LVEF: left ventricular ejection fraction; cTnI: cardiac troponin I; hs-CRP: high-sensitivity C-reactive protein; ET-1: endothelin-1; NO: nitric oxide; CD18: cluster of differentiation 18; HSP70: heat shock protein 70; NS: not significant

Study	Biomarker	Outcome	Statistical Result
Shandling 1997 [7]	CPK	~35% lower at 12–24h	p = 0.03
Stavitsky 1998 [8]	CPK	~7.5% lower	p = NS
Dekleva 2004 [9]	LVEF	46.27→50.81% vs 45.54→44.05%	P < 0.05
Alex 2005 [10]	sE-selectin, CD18, HSP70, troponin I	Lower postoperative levels	P < 0.05
Li 2018 [11]	hs-CRP, ET-1, NO	↓hs-CRP, ↓ET-1, ↑NO	P < 0.05

Heterogeneity across studies was considered substantial based on variability in patient populations, clinical settings, HBOT protocols, and timing of biomarker assessment. Formal statistical heterogeneity measures (e.g., I²) could not be calculated due to the absence of a pooled quantitative analysis.

Discussion

This systematic review provides a qualitative synthesis of clinical evidence assessing HBOT's impact on myocardial injury and inflammatory biomarkers. Overall, findings suggest that HBOT may attenuate biochemical markers of myocardial damage and modulate inflammatory responses, particularly in acute ischemia, perioperative settings, and chronic CAD.

The observed biomarker improvements align with established HBOT mechanisms. Beyond enhanced oxygen delivery, HBOT attenuates ischemia-reperfusion injury through antioxidant enzyme upregulation, neutrophil adhesion inhibition, and NF-κB-mediated cytokine suppression [12]. These pathways explain both reduced cardiac enzyme release and concurrent inflammatory marker improvements documented across studies. Reduced soluble E-selectin and CD18 indicate decreased endothelial activation and leukocyte adhesion critical steps in reperfusion injury [13]. Lower HSP70 signifies attenuated cellular stress responses. In chronic CAD, hs-CRP and endothelin-1 reductions with increased NO suggest improved endothelial function. LVEF improvement observed in some studies suggests potential functional benefit, though translation to hard clinical outcomes remains unproven.

Importantly, these biomarkers have established prognostic value. An individual patient data meta-analysis demonstrated that elevated high-sensitivity troponin T is independently associated with increased mortality and cardiovascular events in chronic heart failure [4]. High-sensitivity CRP remains a well-validated marker of residual inflammatory risk [5], and a 2025 multicentre cohort study confirmed the predictive value of the hs-CRP/HDL-C ratio for cardiometabolic multimorbidity [14]. Elevated IL-6 levels following STEMI are associated with larger infarct size and adverse outcomes [15,16]. Thus, HBOT-induced biomarker reductions are biologically plausible, but whether they translate to clinical benefit requires confirmation in adequately powered trials. Our findings extend a Cochrane review that found insufficient evidence for routine HBOT in acute coronary syndrome [17]. A recent systematic review on HBOT in cardiovascular surgery similarly found potential benefits but called for larger prospective trials with standardized protocols [18].

The small number of studies limits generalizability. Heterogeneity existed in protocols (2.0-2.4 ATA; 1-24 sessions), biomarker timing, and assay methodology. Moderate risk of bias in several studies and incomplete dispersion measure reporting precluded meta-analysis. Publication bias cannot be excluded. HBOT is generally safe, with barotrauma in 1-2% of patients [18]. Current evidence suggests HBOT may reduce myocardial injury and inflammatory biomarkers in selected cardiac populations. Given the strong prognostic value of these biomarkers, these biochemical changes are biologically plausible. However, heterogeneity, methodological limitations, and small sample sizes prevent definitive clinical recommendations. Large, well-designed prospective trials are needed.

## Conclusions

In conclusion, current clinical data suggest that HBOT may exert beneficial effects on the extent of myocardial injury and in dampening systemic inflammation as reflected by reduction in cardiac and systemic inflammatory biomarkers in selected cardiac populations. However, heterogeneity and methodological limitations of existing studies prevent definitive clinical recommendations. Future large-scale, well-designed prospective trials focusing on standardized biomarker assessment and long-term outcomes are warranted to further specify the role of HBOT in myocardial injury management. 
